# Smart Working and Well-Being before and during the COVID-19 Pandemic: A Scoping Review

**DOI:** 10.3390/ejihpe11040108

**Published:** 2021-11-26

**Authors:** Leda Marino, Vincenza Capone

**Affiliations:** Department of Humanities, University of Naples Federico II, 80100 Naples, Italy; vincenza.capone@unina.it

**Keywords:** smart working, well-being, work engagement, technostress, scoping review

## Abstract

The purpose of this scoping review focused on the relationship between smart working, a conception of job centered on the flexibility and autonomy of the worker, and well-being/illness in an organizational context before and during COVID-19. The literature review, conducted using the PRISMA (Preferred Reporting Items for Systematic Review and Meta-Analysis for qualitative synthesis) method for qualitative synthesis, considered studies published from 2014 to 2020. From the analyses conducted by three independent coders, three main areas of interest in the literature emerged: (1) smart working and work engagement, (2) smart working and technostress, and (3) mediators of the relationship between smart working and well-being. The review highlights the need for an organizational culture increasingly oriented towards agile working practices in conjunction with organizational support and training.

## 1. Introduction

The practice of employees working remotely, away from the conventional workplace, has become a varied and fast changing phenomenon [1]. Processes of redefinition of work, already in place in the pre-pandemic era (before 11 March 2020 when the World Health Organization declared the COVID-19 disease a pandemic), with the advent of COVID-19 have become widely required [2]. Different organizations (i.e., tertiary, education, professional services, public administration) became involved in smart working. Specifically, researchers [3] sustain that in the public sector, there was a generalized and indiscriminate use of smart working without understanding, really, the requirements to introduce the smart working experience more than in the private sector, that further improved smart working practice.

The increasing use of new technologies and the progressive automation of work [4] favor the emergence of new organizational paradigms, such as the reduction of days of presence at work to facilitate environmental sustainability and the reconciliation of work and lifetimes, without this leading to a decrease in either productivity or salary terms. We cannot ignore this phenomenon in the Work and Organizational perspective of psychology, such as our field.

Therefore, flexible forms of work are needed, assisted by technological tools that allow work to be carried out in the workplace. Furthermore, “the new ways of working can be an occasion to rethink the company strategy toward a smart working model, rethinking the office space toward downsizing. It can be advantageous in terms of economic return for the firm (fewer square meters occupied amount to less costs) and increase environmental sustainability and better workers’ satisfaction, well-being, and quality of life” [5] (p. 195). 

Smart working refers to a form of work characterized by the absence of time or space restrictions and an organization by phases, cycles, and objectives [3].

This vision has been defined as “a new way of operating and functioning of organizations and as an “effort” to regulate organizational experiences on work flexibility that have been tested for some time” [6] (p. 16).

Smart working and/or telework, as an organizational practice in the pre-COVID phase, has become an inescapable strategy to cope with the pandemic emergency (Italian Ministry of Labour and Social Policies report that the smart working mode involved, as of May 2020, 1 million 800 thousand workers compared to 570 thousand in 2019). Smart working refers to a conception of work activity centered on the flexibility and autonomy of the worker rather than on predefined roles and tasks, following an “a-spatial” concept [7]. Compared to telework as a form of activity that allows people to carry out their duties in places and times different from traditional ones, smart working makes use of the most modern technologies available [6]. Telework refers to the use of electronic information and telecommunications tools to provide remote work. Smart working is a model of work that uses new or existing technologies to improve performance. Telework is becoming a useful invaluable tool during the first phase of the COVID-19 pandemic. However, because organizations had already begun to introduce smart working before the pandemic, it is plausible to assume that they decided to evaluate these procedures for the continuation of the emergency. 

This scenario generally leads the organization to face transformations both at the structural and strategic levels. The literature defines this process as “change management” [8], emphasizing the role that management and organizational “culture play in the transition path in terms of new planning of practices, different direction of spaces and schedules, but also different relational modes with colleagues and end-users” [8] (p. 42) [9].

However, existing empirical evidence on the association between flexible working practices (including teleworking and smart working) and employee well-being is not decisive [10].

Studies prior to the pandemic affirmed that organizations and employees needed to be aware of the benefits and drawbacks of remote working practices due to the growing use of technology and the consequent increase in flexibility. Remote working and some dimensions of organizational well-being (affective state, social, and professional life) were linked positively and negatively. Still, the literature highlighted a greater consensus towards a beneficial impact of this working arrangement [11]. 

Conceptualizations of well-being at individual levels can be categorized on two dimensions [12,13,14]: well-being as a context-free (e.g., general mental health) or as a domain-specific concept (e.g., job satisfaction, work engagement). Following this, the authors suggested that considering the two conceptualizations of well-being was preferable [15]. When well-being was examined as a domain-specific concept, the associations with its antecedents were stronger [14]. 

A part of Reference [16] considers performance related to job conditions, assumed to be associated with poor well-being. Such job conditions include, for example, low work autonomy, too many demands, and lack of social contact and support from colleagues, as is possible during a smart working experience [11]. In fact, Reference [3] identifies some essential requirements about smart working, for example, “replacing the logic of performing tasks with achieving objectives, allowing everyone to manage work actively and autonomously that are important to better performance. So, it is then important to understand how individual behavior contributes to workgroup and organizational performance” [17,18] (p. 308).

However, when considering context-free well-being, we can better capture the mental health of employees and the impact of work on all walks of life.

From an organizational perspective, as suggested by Zeike et al. [19], the choice of the Job Demands-Resources Model (JDR) [20] is stimulating as a broader theoretical framework to explore managers’ working conditions and specific work resources in the context of digitalization, considering the same digital leadership as a resource aimed at positively influencing the psychological well-being of managers engaged with onerous demands related to digital transformation. In the JDR, all the variables involved in a workplace context can be clustered as job demands and job resources [20]. JDR is a transactional model that has been used to examine a variety of working environments or professions. Job demands are defined as “physical, psychological, social, or organizational aspects of the job that require sustained physical and/or psychological (cognitive and emotional) effort or skills” [20] (p. 312). Bakker and Demerouti [20] further defined job resources as “physical, psychological, social, or organizational aspects of the job that are either functional in achieving work goals, reducing job demands and the associated physiological and psychological costs, or in stimulating personal growth, learning, and development” (p. 312). 

During the COVID-19 pandemic, many public and private organizations requested their employees to work from home [21,22]. This has, and presumably will continue to, significantly impacted workers, work–family balance, and work design, which needs to be better understood and analyzed. Before the emergence of COVID-19, the data on the introduction of smart working in organizations referred to small numbers of workers [23]. 

Today, while acknowledging its advantages and disadvantages, companies and employees themselves seem willing to cultivate remote working practices even when the emergency is over [24,25].

### Aim of the Review

Organizational life, especially since the first lockdown due to the pandemic, is experiencing a moment of epochal change that is still ongoing and determines new requirements to be met. 

Such reorganizational aspects, as will be seen, are not the result of the emergence of COVID-19 alone. It diffusely brought to light the organizational change that was already affecting organizations [25] in the postmodern era [26].

The purpose of the following scoping review [27] was to systematize the main studies that, from 2014 to 2020, investigated the relationship between the introduction of remote work practices with individual well-being, taking into account any differences brought about by the pandemic scenario. We adopted the JDR Model variables in terms of resources (for example, organizational support) and demands (for example, technostress) for our research strategy.

## 2. Materials and Methods

The analysis process followed a top-down approach [28]. During the second COVID-19 pandemic wave in Europe (the second wave began on December 2020, and resulted in about 1.2 COVID-19-related deaths per 100,000 per day, a rate almost equivalent to the first wave, which resulted in about 1.35 COVID-19 deaths per 100,000 per day [29]), available elements, such as the constant updating from reliable sources about the use of different modes of remote practices depending on the type of organization, connected to previous knowledge about smart working as a factor related to the reorganization of work contexts and activities, gave rise to inferences shared among the authors, leading to the development of some research hypotheses that are the basis of the objective of the review [28].

The use of the JDR Model and smart working literature [19,30] led to the formulation of some research questions:

1. What are the drivers of job well-being and strain?
2. How do the employees perceive smart working implications on their well-being?
3. What is the role of the pandemic situation in smart working practices, how does it impact the performance and other organizational outcomes?

These research questions propaedeutic to the literature search have followed the PICO strategy [31,32], defining:

(a) The target population, i.e., workers and organizations in smart working.
(b) The focus of the exposure, well-being, and factors of discomfort.
(c) The outcomes, in terms of outcomes related to smart working.
(d) The study designs, in quantitative and qualitative terms.

All four questions are driven by our motivation to contribute to a profound understanding of smart working strategies and the effect on individual and organizational well-being that might inform research and evidence-based programs to support employees’ optimal professional functioning during and after the pandemic. Structured information about the main research fields can facilitate understanding of the meaning of smart working in various organizations. 

Once the field of investigation was defined, the electronic records were identified utilizing a bibliographic search conducted by inserting the algorithm of keywords and Boolean operators such as “AND”, “OR”, and “NOT” in the scientific databases taken into consideration (SCOPUS, EBSCO, and PsycINFO), which were chosen for their reliability and relevance to the social sciences. The study was conducted according to the principles of systematic reviews following the guidelines of PRISMA (Preferred Reporting Items for Systematic Review and Meta-Analysis for qualitative synthesis) [33].

The research was carried out from November 2020 to January 2021 (date of last research: 10 January 2021) and included qualitative and quantitative empirical studies, crossing keywords such as “smart working”, “remote working”, “well-being”, “stress”, and “engagement”. These keywords were chosen to meet the research objectives, i.e., the psychosocial fallout in terms of well-being (“well-being”, “engagement”) and discomfort (“stress”) in relation to smart working (“smart working”, “remote working”).

The inclusion criteria for the selection of articles were only English and Italian language of publications, keywords, title, and abstract always present in English in the case of Italian editions, peer-reviewed journals, the specific target of smart working, clearly defined and explicit methods, reliable detection or clear intervention procedures, presentation of results, the presence of applicative spin-offs, and studies published from 2014 to 2020.

This temporal range was circumscribed by considering the year in which the first bill containing the provisions for the promotion of flexible and simplified forms of telework of 29 January 2014 was filed for Italy.

The exclusion criteria for selecting articles were not being an organizational or psychosocial theoretical perspective (for example, clinical perspective), article review, grey literature articles, letters to editors, conference abstracts, and dissertations.

No geographical restriction was included. 

The search initially identified 60 articles. In the end, three independent coders, experts in the psychology of work and organizations, have analyzed titles, abstracts, and keywords proceeding to the first exclusion process. 

This process occurred twice and involved the coders in each consensus session [34], conducted by videoconference, to reach an agreement about the thematic core concepts that were being formed. At the end of this phase, 15 papers were found to meet the selection criteria.

**Figure 1:**
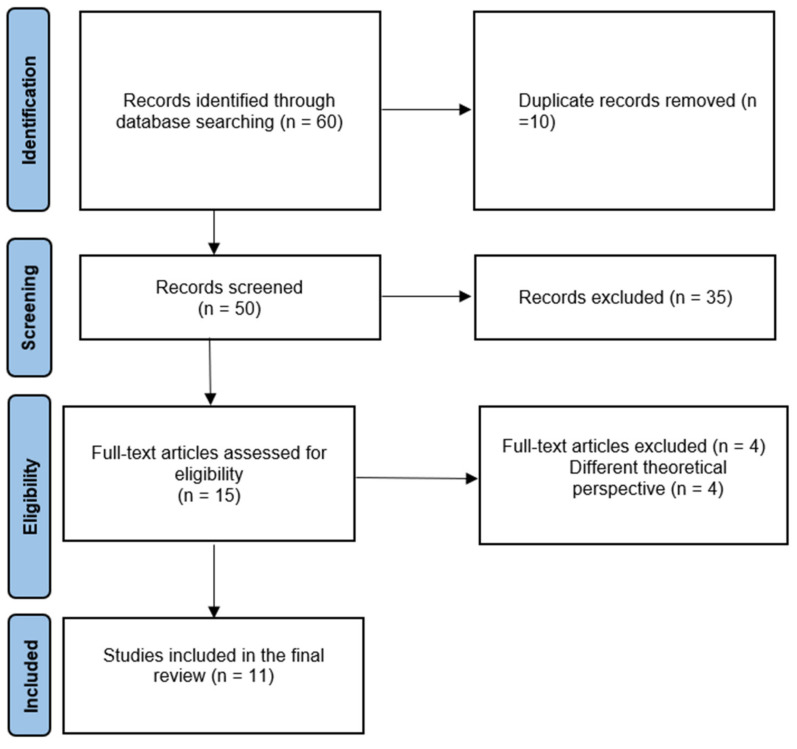
Flow diagram of the literature search strategy and review process [33] (Adapted with permission from PRISMA 2020).

Subsequently, the full-text analysis of each selected article was carried out, proceeding to the further elimination of 4 records. Among the reasons for exclusion were: non-organizational theoretical perspectives, and studies focused on the characteristics of smart working without investigating the relationship with any type of variable. This resulted in a final corpus of 11 articles (see Figure 1 for the flow diagram).

The categories, generated ex-post based on the content analysis of the selected articles, led to three areas of grouping that emerged, mainly on the identification of the most recurrent themes and constructs and the differences between them.

Articles published in the following journals were selected:

Asia Pacific Journal of Human Resources, Employee Relations (2), Frontiers in Psychology, Indian Journal of Commerce and Management Studies, International Journal of Environmental Research and Public Health, Journal of Emerging Technologies and Innovative Research, New Technology, Work, and Employment, Procedia Computer Science, Sustainability (2), and Work and Employment.

## 3. Results

Three main thematic areas were identified: (1) smart working and work engagement, (2) smart working and technostress, and (3) mediators of the relationship between smart working and well-being. In agreement with the literature [35,36], we distinguished well-being, referring to the state of psychophysical health of the worker, from work engagement as the emotional involvement of the workers towards their work [37].

Table 1 presents and summarizes the studies related to each core concept that emerged, reporting for each study the authors, editorial location, description of objectives and participants, method and tools, and the main results.

### 3.1. Area 1: Smart Working and Work Engagement

Studies in this area [38,43,46] have focused on work engagement [45]. The organizations adopted smart working practices both during the COVID-19 pandemic [38] and before [43], as well as making them traditional on par with other classic work modes [43].

Manuti et al. [38] have pointed out, in the pandemic era, how the ability of human resources to involve workers can have a positive effect on their organizational engagement, in line with the literature [41], even when smart working becomes an emergency practice. The use of participatory actions to support changes in working methods and procedures required of employees solicits positive feelings without this appearing to be imposed “from above”, but as a product of their commitment.

Implementing this type of practice in the introduction of smart working has meant that the worker perceives the change as positive and desirable. Connected to this process is the promotion of effective coping strategies [40], that can mediate the reaction between individual resistance to change and the implementation of organizational change. Additionally, Manuti et al. [38] have supported the central role human resources (HR) have in strengthening coping strategies for organizational change related to the introduction of smart working.

The role of human resources has an indirect positive relationship with employee engagement and their extra-role behaviors [42], understood as positive actions not explicitly related to the job task, with a mediating role played by coping strategies. When workers perceive HR involvement in decision-making and change management, their engagement levels increase, as does their positive behavior towards the organization, protecting them from periods of criticality, such as the introduction of smart working in a pandemic scenario, without a precise design of the practice.

Involvement and participation turned the emergency into an opportunity for growth.

In addition, positive attitudes and openness to change were influenced by the acquisition of proactive coping strategies capable of conveying a positive image of change and innovation. Those practices focused on rewards, performance management, worker autonomy, and involvement in task execution were particularly effective.

In the pre-pandemic era, Timms et al. [46] investigated the relationship between smart working, work engagement [45], malaise, and organizational culture [47] and their effects on the lives of employees in the organization. The results showed that an organizational culture perceived as “distant” negatively affected the implementation of smart working and that high levels of engagement, fostered by the perception of a supportive organizational culture, were present in the process of implementing flexible working.

Additionally, about employees’ mental health, the organizational culture played its role when supportive, protecting against symptoms of stress, anxiety, and depression [51]. Being married and having children was correlated with higher levels of engagement in the pre-pandemic phase. Conversely, experiencing work overload was associated with turnover intentions, especially when the organization did not recognize the effort put in by employees and did not support them [46].

Rana et al. [43] investigated, in the pre-pandemic era, the relationship between smart working, engagement, and effective performance. The study showed significant correlations between the dimensions of engagement and job performance, leading to organizational goals and positive employee behaviors; in addition, employees’ commitment to remote work activities emerged as a predictor of effective performance [44]. Specifically, dedication had a strong relationship with contextual performance, as did vigor with task performance. Concerning counterproductive performance, these had very weak relationships with the three dimensions of job engagement.

### 3.2. Area 2: Smart Working and Technostress

Studies in this area [52,56,60] have focused on technostress, a particular form of work-related stress linked to the use of digital devices and ICT [41], as a cause of malaise for smart workers, leading to lower productivity and high costs for the organization [52].

Technostress is articulated in the proposed studies in different dimensions: (1) Technological overload, whereby people perceive a discrepancy between their work rhythms and the times dictated by technology and may ill-adapt to changing work habits (techno-overload). (2) Technological invasion, i.e., the perception that the boundaries separating the work context from private life are no longer clear and sharp (techno-invasion). (3) Complexity, due to the use of technologies, i.e., those situations in which the worker perceives his skills as inadequate in the face of the performance of a task carried out with the use of ICT (techno-complexity). (4) Insecurity, deriving from the use of technology when this is perceived as a threat to job stability and a factor contributing to the loss of one’s job (techno-insecurity). (5) Uncertainty, given the perceived disorientation in the face of constant changes in the world of ICT and the consequent need for continuous learning and training experienced as a disturbance in their work or as a “constraint” necessary to continue in the work (techno-uncertainty) [57].

In the pandemic period, Molino et al. [52] found positive and significant correlations between the dimensions of technostress and two main outcomes, namely work-related stress and work–family conflict, during the first Italian lockdown. In particular, researchers have found an increased risk of stress in smart workers who had a high workload, confirming that the technostress is related not only to the use of ICT but also to how the comparison between the individual and digital devices occurs (for example, time of use and workload-associated).

The study by Spagnoli et al. [60] found a relationship between workaholism and technostress again in the pandemic period. Furthermore, the interaction between workaholism, authoritarian leadership, and remote work significantly affected workers’ levels of technostress. Concerning gender, women experienced higher levels of technostress than men.

The relationship between smart working, technostress, and work-related stress was investigated in the pre-pandemic era by Oh and Park [56]. They analyzed the role of some variables such as job satisfaction, work–life conflict (conflict between work and personal life) [43], and prolonged work time in the onset of technostress.

The results showed an indirect negative effect of technostress on job satisfaction, and among the antecedents of technostress was overexposure to technology use by workers.

Higher levels were present among those who, while working remotely, consulted applications and instant messaging during and after work and checked e-mail during recovery periods [56].

Moreover, the relationship between technostress and job satisfaction was mediated by work–life conflict, while technostress and work–life conflict were strongly correlated.

Thus, both at the time of COVID-19 and in the pre-pandemic era, work overload, overuse of digital devices, and complex management of personal and family lifetimes contributed to increased levels of technostress among smart workers [52,56].

### 3.3. Area 3: Mediators of the Relationship between Smart Working and Well-Being

Studies in this area [19,63,64,65,67] regarded the mediators of the relationship between smart working and psychosocial well-being, and almost all of them refer to the pre-pandemic period. Of particular note are work–life balance [63,64,65], digital leadership styles [19], and the pandemic context [67].

Felstead and Henseke [63] found that worker autonomy concerning the possibility of choosing whether or not to adhere to remote working practices acted as a mediator between the introduction of smart working in the company and well-being, identified as job satisfaction and affective commitment.

Grant et al. [64] found that personal and organizational competencies played a mediating role between balance and well-being with smart working. Employees needed to perceive that the organization has confidence in completing work on time and with the required quality. They emphasized the need for an organizational culture based on trust and to have the same access time to software as managers, so they could manage their work without being constantly monitored. Experiencing autonomy was an essential aspect of smart working. Employees who experienced less autonomy preferred to work in the office, while managerial levels, having access to the full range of technologies, could flexibly manage working hours. One challenge was concentration.

Some interviewees stressed using skills for not being distracted by external factors that are not usually present in the workplace. Relevant skills related to self-management, such as knowing when to stop and take breaks to counteract the sense of “dependence” on the computer left running even after working hours, disturbing sleep–wake rhythms [64].

Adaptation of one’s behavior emerged strongly, therefore, also concerning family life, leisure, and social time. Workers had to develop strategies to maintain good levels of balance. In addition, it was found that work pressure increased for those who, for example, managed more responsibilities. At the same time, decision-making did not seem to be affected by remote working, either in terms of the quality of decision-making processes or the amount of decision-making space for employees facilitated by access to more information in real-time.

The increased productivity of remote workers was linked to working for long periods in silence, on large files, completing work even in a single day, and circumventing the social processes that took time away from activities when in attendance.

Work–life balance and boundary management were closely linked to well-being. Remote working improved the ability to work flexibly. The time spent out of the office had a positive effect both for the employer and the employee in terms of reduced commuting, and reduced stress related to travel and doing household chores in a more organized way. Conversely, the issue of health emerged strong with the management of boundaries, although some had developed an unhealthy ability to work 24 h or seven days continuously.

Finally, determinants of well-being were effective organizational communication and maintenance of this with colleagues, relationships outside working hours, and support from members of their group and interactions with family members.

Regarding mental well-being, the study by Grant et al. [65] found that this was significantly correlated with work productivity, experienced trust in the organization, and better work management in terms of flexibility. Conversely, remote working could lead to ‘interference’ with private life with adverse outcomes for overall health status [66].

Concerning the pandemic scenario, Prasad et al. [67] analyzed the relationship between smart working and occupational stress, taking into account variables such as gender and age. The results did not identify gender as a mediating or moderating variable.

Variables that mediated (negatively) the relationship between well-being and smart working included isolation from co-workers, distractions from family, lack of suggestions about work practices, failure to balance work time, and poor ability to design work independently. On the other hand, positive mediators were the effective management of work flexibility, a greater degree of control over one’s work, higher levels of role autonomy, and the use of new technologies per se.

Finally, this area included the study of Zeike et al. [19], who investigated whether the perception of adequate digital skills by management (upper-level managers) to guide organizational members in the use of technology impacted their levels of psychological well-being. The study highlighted that the perception of having adequate digital leadership skills leads to higher levels of psychological well-being when choosing to introduce smart working in the organization.

## 4. Discussion

Today, organizations are called upon to adopt a mixed approach, which integrates the analytical dimension related to the evaluation of costs and productivity, and the human factor with its needs, potential, and the presence of soft skills that can be activated even within different configurations of the work environment and its time [22]. First of all, the review has highlighted the importance of applying a person-centered perspective for the development of internal policies in the organization that, at the same time, considers the parameters of effectiveness and efficiency related to the performance of the individual, of the group and of the company’s productivity [38]. 

Indeed, the implementation of smart working requires adequate management policies with particular attention to the reorganization of work and personnel management [3,72]. It would be necessary for organizations to utilize human resource management more effectively, implementing a more inclusive and differentiated approach to support employees, and thinking about the workers’ different needs to better balance work and private life [30]. The digitization of work processes and tools is not a mechanical and automatic process. For example, today, it is common in organizations to socialize with colleagues both personally and electronically [11]: it requires parallel development of a “digital culture” [73] that presupposes these tools’ virtuous, conscious, and critical use.

The latter is, moreover, a constituent element of the organization itself and quite complex to analyze. Corporate culture moves or hinders change. The literature has highlighted how, especially in the face of health emergencies and environmental disasters, the challenges of the organizational system can only be met with the involvement of the whole system, and that the organization’s resilience is based on factors such as leadership and organizational culture. Therefore, different factors must be considered in a phase of change, ranging from communication management to respect for health and safety, not forgetting work–life balance and performance, especially when the uncertainty during a pandemic has compromised people’s planning abilities about the future, leading to the phenomenon of “pandemic fatigue” [74].

Workers, after all, must be adequately trained and have adequate resources to be able to offer good performance [38]. The literature [30] about the JDR Model and smart working demonstrated that job demands (workload and social isolation), organizational job resources (perceived organizational support), and individual job resources (self-efficacy, vision about the future, and commitment to organizational change) have affected workers’ quality of life during the pandemic. Whoever manages the human resources of an organization is therefore called on to implement new practices, aimed at reinforcing the workers’ engagement even when the work methods change, motivating them to adopt proactive behaviors and positive attitudes, achieving results in line with the expectations of the organization [38]. From a preventive perspective to introduce remote work, especially in a period of crisis such as the pandemic, the organizational support to increase workers’ resources could be a tool to be implemented to prevent and preserve well-being.

Furthermore, different companies have activated training and education paths focused both on the use of digital technologies and the acquisition of greater autonomy by employees and delegation and coordination skills by leaders [7], processes considered basic for smart working practices to be effective.

Indeed, as a resource, organizational training played a vital role in coping with change [46] and was one of the first elements to take advantage of the combined and smart philosophy, thanks to the pioneering use of digital technologies and internet connections for training purposes [21]. However, not only organizational resources, but also the mediating role of coping strategies [38] is relevant, which significantly impacted employee engagement, highlighting the need for organizations to pay special attention to the promotion of effective coping strategies to support employees during organizational change [75]. 

Human resources [38], whose role can facilitate the transition towards remote working practices by privileging clear information congruent with workers’ expectations, can become a strong lever towards accepting the new practices. 

Additionally, considering the relationship between organizational actors to achieve well-being outcomes, communication is essential in the service of the organizational process and its containment function for internal users [76]. Communication could help to reduce the sense of isolation and insecurity experienced by employees, especially when remote working is the only possible option, as in the emergency period [77]. Communication is also part of organizational culture [78].

For those who deal with organizational psychology, organizational culture means the founding engine of the organization. This review highlighted the role of organizational culture to promote flexible work, with positive effects on work performance and engagement, in line with the literature [37] about work engagement that highlighted the role of positive feedback to help workers to manage and improve their performance. Good performance in the context of work engagement [43] also seems to emerge from engagement in work activities. It is not surprising, because although engagement and work engagement are two distinct constructs [37], which do not indicate that in the presence of one, there is also that of the other, they can influence each other [79]. Underlying the “digital culture” are values such as openness to innovation, cooperation, flexibility, and the ability to delegate, as well as managerial strategies marked by “agility”, in line with the study [64], which have highlighted the negative feelings of smart workers in seeing an organizational culture oriented to continuous digital monitoring.

Finally, as for the discomfort experienced by workers in smart working, it has emerged that technostress needs particular interventions for prevention [52]. Smart working, tending to increase the use of technology, pushes some workers to make excessive use of it, even beyond working hours, with the risk of exacerbating conflicts in the family and personal life [56]. Therefore, when planning and implementing smart working practices, it is necessary to consider those factors that can contribute to lowering the levels of this particular work-related stress to avoid consequences on workers’ health status, organizational well-being, and not least on performance [52,56,60].

## 5. Conclusions

The introduction of smart working should facilitate management and work autonomy, to reduce stress levels and pressures experienced in the workplace [80].

However, the contingency factor related to the COVID-19 pandemic has entailed costs for individual workers: a significant expenditure of cognitive energy, necessary to learn new procedures and modify their work routines related to maintaining pre-pandemic phase productivity [52]. Technostress, as a health risk factor, became more acute as workers were intent on “reconverting” their work and personal lives by becoming smart and remote workers.

Smart, or agile, work by definition does not involve precise time (and place) constraints. Concerning managing work time and space, this review study has emphasized the role of work–life balance [63,64,65] as a promoter of well-being and adequate performance. Organizations need to design work so that agile does not turn into permanent returns. Smart workers need to be able to experience a supportive culture [46], which allows them to adequately manage the boundaries between personal and work life [81]. 

Within their roles, managers and leaders can play a decisive role in fostering employees’ work well-being, counteracting the loss of confrontation with their colleagues experienced by smart workers with clear information about the objectives of the task and the ways through which it should be performed [19,60]. Indeed, the literature [82,83] has shown how it is necessary to focus on “capacity building of administrators and managers, so they become more familiar with e-learning tools, and usage of innovative technology, […] high level of emergency preparedness is also needed, and this preparedness will require source allocation to deal with individual well-being challenges” [82] (p. 164). In addition, although the effects of leadership styles for workers’ well-being have not been investigated in the proposed studies, it seems that a non-authoritarian leadership style can contribute to lowering the workaholism and technostress of organizational members.

The function of individuals’ abilities, beyond the role, in changing behaviors and adapting to the “agile” transition emerged [63]. While some have prolonged work activities by taking time away from family and personal life, others have more peacefully taken time away from work by devoting more time to family in a self-management logic [67].

From the analysis of the studies, the role of organizational commitment [84] and enjoyment in carrying out one’s work emerged, and the authors of [11] had already shown how “the sense of trust in that individuals will appropriately conduct their work duties outside an office environment can increase individuals’ organizational commitment” (p. 69). Smart working has influenced belonging to the organization, and the quality of this bond, when it has been a choice made by employees, supported by their motivation, and/or when it has been intentionally implemented by the organization, transferring trust, seeking alliance and participation from the members of the entire organization [85]. For example, organizations should be encouraged to create social support networks between remote e-workers, colleagues, and supervisors. Good communications between remote e-workers and their office-based colleagues need to be encouraged, especially when task interdependence is involved. “Effective planning of remote e-workers’ office presence could be a useful coping strategy” [11] (p. 69).

Nevertheless, the relationship between remote work practices and well-being is still an open debate [11]. Some scholars [7] believe that high levels of work flexibility and autonomy related to effective communication and good work–life balance are elements that contribute to high levels of work well-being. Other studies [86] found that remote working negatively influenced employees and over-intensifies work rhythms, creating levels of stress. However, as highlighted in this review, they were linked to an over-use of technology and “blurred” boundaries between personal and work life. The literature [11] considers that there is insufficient empirical evidence for organizations, management, and human resources such as to conclude that remote working is beneficial to the psychosocial well-being [87] of employees. In view of a health emergency still in progress, it will be interesting, in future developments, to study and analyze the reorganization of work in an era that, soon, will be “post-pandemic.”

This review has several limitations. The keywords did not always make studies consistent with the subject areas and may suffer from a criterion of subjectivity, which in subsequent studies, should be controlled more [88]. In the studies, the prevalence of the cross-sectional method and the different geographical areas do not allow for generalizable results. In addition, given the small amount of work that emerged, we chose to include those related to “remote working”, being aware that the latter refers to the performance of work from home rather than the broader parameters of decision-making autonomy, flexibility, and a-spaciality of smart working [21]. There is a lack of statistical indices of agreement detection on the identified thematic categories, which would have strengthened the methodological framework of the work.

However, despite the inevitable limitations, this work is a first analysis of the critical points in terms of the study of psychosocial factors related to the introduction of smart working in organizations, taking into account the pre-pandemic phases and the subsequent start of an emergency (which was our range of interest), laying the foundations for what may be future policies, especially in organizational terms, to be adopted to deal with the crisis still in progress, but also in future scenarios of work reorganization. This review suggests that the changes related to work in this era involve different fields of study and that future research could be focused on: (a) a deeper understanding of organizational culture and how to implement a digital culture, (b) a greater understanding of the use of ICT and their influence on workers, especially when they become a source of malaise with a phenomenon such as technostress, (c) organizational practices of prevention and promotion of resources to cope with changes related to working methods, and (d) a multidimensional approach in which academics and managers consider theories and field experience to further improve the understanding of a phenomenon that, today, involves all types of organizations, from universities to industries.

## Figures and Tables

**Table 1:** Description of included studies.

Authors/Year	Title/Journal	Aim of Study	Participants	Type of Study/Methods	Instruments	Main Results
Area 1: Smart working and work engagement						
Manuti, Giancaspro, Molino, Inguisci, Russo, Signore, Zito and Cortese, 2020 [38]. CP	“Everything Will Be Fine: A Study on the Relationship between Employees’ Perception of Sustainable HRM Practices and Positive Organizational Behavior during COVID19. Sustainability	To detect workers’ engagement in human resource management practices and their coping strategies towards organizational change.	549 Italian employees, among them 40.3% were employed in the public sector and 49.5% in the private sector. Of whom 62% were F and 37.7% M. 76.3% were married or cohabiting and 57.2% had no children. 71.6% had open-ended employment contracts. 50.3% were employees while 12.8% were managers and executives. Regarding professional sectors: 28.6% tertiary; 16.6% education; 14.1% professional services; 10.6% secondary; 7.1% healthcare; 6% primary; 16% other sectors. 63.9% were in smart working.	Quantitative cross-sectional	Self-report questionnaire consisting of 5 items from the HRMPPS [39]; 3 items from the Coping with organizational change scale [40]; 3 items from the organizational engagement scale [41]; 2 items from the Extra role behaviour scale [42].	Organizational changes perceived as positive increased engagement levels by improving coping strategies. Organizational commitment and positive behaviors outside the job role (extra-role) were positively correlated with the ability to involve human resources and coping strategies. Smart working showed a positive correlation with organizational commitment, extra-role behaviors, and human resources involvement, whose perception was positively correlated with organizational involvement and positively associated with organizational change. The adoption of strategies by workers to promote, disseminate, and support change depended on organizational communication and support in the change process.
Rana, Pant and Chopra, 2019 [43]. PCP	Work engagement and individual work performance: research findings and an agenda for employee relationships. Journal of Emerging Technologies and Innovative Research	To detect the association between engagement dimensions (vigor, absorption, and dedication) and task- and context-related job performance and their relationships.	134 Indian workers (clerks and managers) in the ICT sector, of which 62.7% were M and 37.3% F, with a career seniority of *M* = 11 years.	Quantitative cross-sectional	Online self-report questionnaire consisting of 3 socio-anagraphic questions, the job performance scale [44], and the UWES-9 [45].	Engagement presented significant correlations with individual and organizational performance. Vigor, absorption, and dedication presented a significant relationship with task performance and contextual performance.
Timms, Cook, Brough, O’Driscoll, Kalliath, Siu, Sit, and Lo, 2015 [46]. PCP	Flexible work arrangements, work engagement, turnover intentions and psychological health. Asia Pacific Journal of Human Resources	Detect the correlation between smart working, engagement, psychological distress, and the role of organizational culture in supporting the implementation of smart working.	823 employees from 8 Australian organizations in banking, education, public service, and social services. Of these, 72% F with age *M* = 43, of which 57% with family (married and/or with children). 75% full-time contract and career seniority *M* = 11.	Quantitative Longitudinal	Self-report questionnaire administered in 2 stages (12-month interval) consisting of the Organizational Culture Scale [47], 3 items of the turnover intention measure [48], the Supervisor Support Scale [49], the flexible work organization sub-scale (FW) [50], the UWES-9 [45], and the anxiety/depression sub-scale [51].	A supportive organizational culture in the introduction of smart working increased levels of engagement by protecting against discomfort and turnover intentions. Being married and having children correlated with higher engagement levels. Being single and experiencing work overload (hours) was associated with turnover.
Area 2: Smart working and technostress						
Molino, Inguisci, Signore, Manuti, Giancaspro, Russo, Zito and Cortese, 2020 [52]. CP	Well-being costs of technology use during COVID-19 remote working: An investigation using the Italian Translation of the Technostress Creators Scale. Sustainability	To test the psychometric characteristics of the Italian version of the Technostress Creators Scale (Study 1) and use it in relation to the emergence of COVID-19 (Study 2).	Study 1: 878 Italian workers, 57.7% F and 42% M, with age *M* = 39 years. 53.4% married/cohabiting and 55.5% without children. 56.7% permanent contract. 53.4% were in smart working. Study 2: 749 Italian workers, 58.5% F and 41.3% M, with age *M* = 38.6 years. 51% married/cohabiting and 57.7% without children. 52.5% had an open-ended contract. 62.6% were in smart working for *M* = 4.74 days during the first Italian lockdown.	Validation study, quantitative cross-sectional	Technostress creators scale Italian version [52], 3 items of the workload scale [53], 3 items of the work–family conflict scale [54], and the COPSOQ for behavioral stress assessment [55].	The technostress creators scale was validated for the Italian context. The results of Study 2 showed a positive and significant correlation between stress, work–family conflict, technostress, and workload. Smart working was related to the dimensions of technostress and behavioral stress. Psychosocial malaise related to the pandemic was present, accentuated by smart working.
Oh and Park, 2016 [56]. PCP	A study of the connected smart worker’s technostress. Procedia Computer Science	To investigate the effects of work–family conflict, technostress, and related mitigating factors, and the use of technology beyond working hours on job satisfaction.	345 Korean managers, 51% M and 49% F.	Quantitative cross-sectional	Online self-report questionnaire consisting of Technostress Scale, Technical Support, Promotion of Involvement, Job Satisfaction [57], Work Continuity after Daily Work [58], and Work–Life Conflict [59].	An indirect influence of technostress on job satisfaction, mediated by work–life conflict, emerged.
Spagnoli, Molino, Molinaro, Giancaspro, Manuti and Ghisleri, 2020 [60]. CP	Workaholism and technostress during the COVID-19 emergency: the crucial role of the leaders on remote working. Frontiers in Psychology	To explore the role of authoritarian leadership in relation to administrative employees of a university placed in partial and/or total smart working and to examine associations with workaholism and technostress.	339 Italian university administrators, 46.6% M and 53.4% F, with age *M* = 48 years. 34% held positions of responsibility and 83.5% had a career seniority of *M* = 10 years. 53% were partially in smart working and 47% completely.	Quantitative cross-sectional	Online self-report questionnaire composed of the 10-item Dutch Work Addiction Scale Italian version [61], 6 items of the Toxic Leadership Scale [62], and Technostress Creator Scale Italian version [52].	Workaholism was positively correlated with authoritarian leadership style and technostress. The interaction between workaholism and authoritarian leadership was significantly correlated with technostress. Smart working was not significantly correlated with technostress, nor were the interactions between workaholism and smart working and between authoritarian leadership and smart working, but the interaction between workaholism, authoritarian leadership, and smart working was significantly correlated with technostress, which affected women more, at high levels of workaholism and in the presence of a strong authoritarian leadership.
Area 3: Mediators of the relationship between smart working and well-being						
Felstead and Henseke, 2017 [63]. PCP	Assessing the growth of remote working and its consequences for effort, well-being, and work–life balance. New Technology, Work and Employment	To investigate which types of work are progressively adopting smart working and the implications this has on fatigue, organizational commitment, well-being, and work–life balance of workers.	45.000 British workers over 16.	Quantitative analysis of growth trends in smart working and its implications on workers’ lives using national databases relating to periodic surveys of the UK population.	Labour Force Survey (1997–2015), and Skills and Employment Survey (1981–2012).	From 1997 to 2014, the adoption of smart working presented an increase of 5%, except for places suitable for labor (e.g., factories). Compared to traditional workers, smart workers had a better attitude toward their organization and 70% would not leave their organization for another work setting, reporting high levels of organizational commitment, job enjoyment, and high levels of job satisfaction. However, 44% feared losing their jobs, 39% experienced more fatigue from working beyond their scheduled hours, and most experienced negative effects of smart working on their work–life balance.
Grant, Wallace and Spurgeon, 2013 [64]. PCP	An exploration of the psychological factors affecting remote e-workers’ job effectiveness, well-being, and work–life balance. Employee Relations	Highlight issues related to the impact of remote working on work effectiveness, understood as the evaluation of performance results, work–life balance, and employee well-being. Identify relevant issues about remote working and the implications for managers and employees	3 managers, 4 employees, and 4 administrative staff from 5 public and private organizations in England, of whom 4 were M and 7 were F. Of these, 5 had children and 2 were careers of elderly/non-self-sufficient people.	Qualitative cross-sectional	Semi-structured interview (between 40 and 90 min) aimed at the three macro-areas of investigation and administered in person, by phone, and by e-mail, consisting of 7 sections: (1) biographical data sheet, (2) job role. (3) technology, (4) practices and (5) measurement of smart working, (6) life and work, and (7) further observations.	The thematic analysis identified 10 themes: (1) remote work practices (digital devices and work activities), (2) work–life balance, (3) social interactions, (4) role autonomy, (5) managing work–life boundaries, (6) decision making, (7) productivity, measurement, and performance, (8) differences, skills, and competencies, (9) adaptive behaviors, and (10) trust. With reference to well-being, support from colleagues and family members, communication, reconciliation of difficulties, and management of social networks emerged as crucial.
Grant, Wallace, Spurgeon, Tramontano and Charalampous, 2019 [65]. PCP	Construction and initial validation of the E-Work Life Scale to measure remote e-working. Employee Relations	Develop and validate the EWL measurement scale for smart working based on the study by Grant et al., 2013.	2 independent samples: (1) 250 workers from 11 UK public and private organizations, 63% were F and 37% M, with age range 24–54. 73% were professionals and managers with smart working experience =/> 2 years. (2) 219 English university employees, 66% F and 34% M, with age range 25–54. 77% had full-time contracts, 14% smart working experience > 10 years.	Quantitative cross-sectional	Online self-report questionnaire, consisting of biographical section, ad hoc items on job role and ICT use, open-ended questions on work–life balance, EWL Scale [66], 3 subscales (GH, VT, MH) from Health Survey SF-36v2 [50].	The validated scale was aimed at organizations intent on promoting smart strategies by supporting employee well-being, identifying barriers and facilitators, and assessing the impact of technology on employee well-being. Four main areas emerged: work effectiveness, relationship with organizations, e-well-being, and work–life balance measured through productive effectiveness, organizational trust, flexibility, and work–life interference through 28 items on a 5-point Likert scale.
Prasad, Mruthyanjaya Rao and Vaidya, 2020 [67]. CP	Effect of occupational stress and remote working on psychological well-being of employees: an empirical analysis during the COVID-19 pandemic concerning the information technology industry. Indian Journal of Commerce and Management Studies	To evaluate the effect of work-related stress on the psychological well-being of ICT workers during smart working imposed by COVID-19 and to analyze gender and age differences.	400 Indian workers, 60% M and 40% F. Among them, 150 had age range 20–30, 110 age range 31–40, 75 age range 41–50, and 65 age range 51–60.	Quantitative cross-sectional	Self-report questionnaire consisting of registry section, 37 items related to work-related stress [68], and Psychological well-being scale short version [69].	Work-related stress significantly affected psychological well-being during COVID-19. It was influenced by the presence of colleagues, role ambiguity, organizational climate, and job satisfaction. Differences in gender and age group were insignificant. Smart working had challenging aspects, such as social isolation, family interference, absence of colleagues, and lack of organizational support. Advantages included flexible working hours and the use of new technologies.
Zeike, Bradbury, Lindert and Pfaff, 2019 [19]. PCP	Digital leadership skills and associations with psychological well-being. International Journal of Environmental Research and Public Health	To develop and test a measurement tool on managers’ perceived digital leadership skills and explore whether these skills were associated with psychological well-being.	368 top managers of a German ICT organization engaged in corporate reorganization, 77% were M, 23% F. 47% range age 41–50 years.	Quantitative cross-sectional	Online self-report questionnaire consisting of WHO-5 Well-Being Index [70], Digital leadership skills scale [71] in 6 items on a 5-point Likert accord scale, and managerial experience indicator in years [72].	The scale was tested and a significant correlation was found between psychological well-being and perceptions of digital leadership ability in managers. 78.5% experienced high levels of well-being.

Legend: F = female; M = male; *M* = average; PCP = pre-COVID-19 pandemic; CP = COVID-19 pandemic.

## Data Availability

Not applicable.
